# Comparative Study of Supervised Machine Learning Algorithms for Predicting the Compressive Strength of Concrete at High Temperature

**DOI:** 10.3390/ma14154222

**Published:** 2021-07-28

**Authors:** Ayaz Ahmad, Krzysztof Adam Ostrowski, Mariusz Maślak, Furqan Farooq, Imran Mehmood, Afnan Nafees

**Affiliations:** 1 Department of Civil Engineering, Abbottabad Campus, COMSATS University Islamabad, Abbottabad 22060, Pakistan; ayazahmad@cuiatd.edu.pk (A.A.); afnan@cuiatd.edu.pk (A.N.); 2 Faculty of Civil Engineering, Cracow University of Technology, 24 Warszawska Str., 31-155 Cracow, Poland; mmaslak@pk.edu.pl; 3 Department of Building and Real Estate, The Hong Kong Polytechnic University, Hung Hom, Kowloon, Hong Kong, China; Imran.mehmood@connect.polyu.hk

**Keywords:** concrete, compressive strength, high temperature, prediction, decision tree, bagging, gradient boosting

## Abstract

High temperature severely affects the nature of the ingredients used to produce concrete, which in turn reduces the strength properties of the concrete. It is a difficult and time-consuming task to achieve the desired compressive strength of concrete. However, the application of supervised machine learning (ML) approaches makes it possible to initially predict the targeted result with high accuracy. This study presents the use of a decision tree (DT), an artificial neural network (ANN), bagging, and gradient boosting (GB) to forecast the compressive strength of concrete at high temperatures on the basis of 207 data points. Python coding in Anaconda navigator software was used to run the selected models. The software requires information regarding both the input variables and the output parameter. A total of nine input parameters (water, cement, coarse aggregate, fine aggregate, fly ash, superplasticizers, silica fume, nano silica, and temperature) were incorporated as the input, while one variable (compressive strength) was selected as the output. The performance of the employed ML algorithms was evaluated with regards to statistical indicators, including the coefficient correlation (R^2^), mean absolute error (MAE), mean square error (MSE), and root mean square error (RMSE). Individual models using DT and ANN gave R^2^ equal to 0.83 and 0.82, respectively, while the use of the ensemble algorithm and gradient boosting gave R^2^ of 0.90 and 0.88, respectively. This indicates a strong correlation between the actual and predicted outcomes. The k-fold cross-validation, coefficient correlation (R^2^), and lesser errors (MAE, MSE, and RMSE) showed better performance than the ensemble algorithms. Sensitivity analyses were also conducted in order to check the contribution of each input variable. It has been shown that the use of the ensemble machine learning algorithm would enhance the performance level of the model.

## 1. Introduction

Due to the fact that concrete has a relatively low cost when compared to other materials, as well as the fact that it is commonly used in engineering structures all over the world, its technology is subjected to constant innovations and improvements [1]. The fast and advanced development of urbanization requires a high demand for concrete [2], which possesses many desired properties including compressive strength, the ability to adopt any shape, and the capacity to resist environmental conditions [3]. In addition, porosity, impact resistance, fire resistance, durability, and acoustic insulation are also cited as being the advantages of concrete [4]. These various aspects enable it to be applied in the construction of infrastructures, dams, tunnels, bridges, and reservoirs [5]. The local availability of ingredients, such as coarse aggregate, fine aggregate, water, and binding material, significantly influences the economical factor [6]. In comparison, other building materials such as steel also possess many properties but cannot be cheaper than concrete. However, in order to make concrete a more advantageous material with improved properties, the techniques of adding other materials such as fly ash, silica fume, other cementitious material, and various fibers are widely adopted [7,8,9]. The use of waste materials in concrete plays a vital role in minimizing environmental risks, as well as in reducing the cost of the material [10]. High temperature and fire severely affect the properties of concrete in both its fresh and hardened states [11]. Some structures or structural elements are exposed to high temperatures, i.e., chimneys, factories with chemicals, and structures used in atomic industries. Moreover, the casting and curing of concrete in hot areas are considered a challenging task to perform, and what is more, concrete loses its mechanical properties (compressive and flexural strength) at high temperature, which ultimately results in the loss of its durability [12].

The development of new materials and methods for protecting against high temperatures has gained more importance in the field of research due to the increased number of incidents caused by fire [13,14]. The effect of fire is considered as a high frequency disaster, which not only causes the deterioration of cement composites, but also plays a role in the spalling of such material [15,16]. The paper [17] indicated that the resistance of a structure against the impact of high temperature caused by fire is one of the critical factors that influence the safety of using structures. This issue requires further research. Concrete is a commonly applied material and is also considered to be one of the best materials for protecting against high temperatures and the effect of fire [18,19]. The components of concrete (at the stage of the hydration of C–S–H and Ca(OH)_2_, and at the stage of the formation of calcium aluminate gels), due to an extended exposure to heat, can disintegrate. This can result in the deterioration of the physicochemical properties of concrete. Therefore, scientists concentrate on analyzing the influence of raised temperature on the mechanical properties of hardened concrete. The differences in the flexural and compressive strength of both ordinary and high-performance concrete have also been investigated when cooled in various conditions (air and water) [20]. In cement composite material (concrete), the decomposition reaction occurs due to the high porosity of the cement matrix and a decrease in strength parameters. The residues of calcium hydro silicate can be recognized in the cement matrix when the material is exposed to high temperatures of about 600 to 700 °C [21]. The performance of other types of concrete, i.e., lightweight concrete, have also been investigated with regards to the impact of high temperature [22]. Extensive research work has been carried out by researchers in order to investigate the mechanical properties of concrete heated to temperatures of up to 800 °C [23,24,25] or higher [26,27,28]. It was proven that the rise in the natural temperature (which depends on the climate zone) also has a significant effect on the properties of concrete, which also involves several energy projects [29,30].

Although concrete is generally a non-combustible material, its chemical, physical, and mechanical properties are directly affected by excessive temperature [31]. Thermal stresses, decomposition, and dehydration cause the spillage, perforation, and cracking of concrete [32]. Moreover, the strength properties of the ingredients of concrete at high temperatures are reduced. Cement paste requires a standard temperature range in order to work effectively inside the concrete matrix. High temperature does not allow cement paste to contribute positively towards the strength of concrete. This is especially the case for high-strength concrete, as it requires a normal temperature to achieve its desired strength [33]. The failure of concrete due to fire is caused by many factors, such as the heating rate and temperature, or structural element conditions, i.e., the application of loads [34]. Therefore, it is usually difficult to analyze the direct effect of high temperature on concrete, especially with regards to the microstructural changes of the aggregate, hydrated cement paste, and interfacial transition zone [35].

Sammy et al. [36] studied the compressive strength and properties of high-performance concrete at high temperatures of about 800 and 1100 °C, as well as during cooling. They investigated that the strength properties decreased sharply after a gradual (26–34%) and rapid cooling process (22–28%). Haruin at al. [37] investigated the effect of high temperature on the compressive strength and splitting tensile strength of light weight concrete with fly ash. The experimental investigation was conducted at 200, 400, and 800 °C. In the case of 800 °C, a decrease by 63.8% and 76.45% in the compressive strength and the splitting tensile strength of concrete, respectively, was noted. Sammy et al. [38] compared normal-strength concrete and high-strength concrete subjected to high temperatures. The 28-day compressive strength of concrete was tested after different exposure times of various temperatures (400, 600, 800, 1000, and 1200 °C). The compressive strength of concrete containing rubber-modified recycled aggregate was also investigated at elevated temperatures [39].

It is clear from past studies that input parameters directly correlate with output results [40,41]. Supervised machine learning approaches also have the capability of incorporating the effect of temperature change, which indicates the positive aspect of these techniques. ML algorithms show a better performance, with a smaller variance, when considering the parameter of temperature change [42]. The performance of ML approaches is associated with several parameters, including the number of parameters and the data that are used to create the model. The novelty of the authors’ research approach also includes the addition of another parameter (temperature effect) for predicting the strength of concrete. The ML approaches, and their comparison in terms of their performance, were investigated in this study. This study included the temperature effect, which was used as an input parameter for investigating the performance of the selected ML approaches during the prediction of the compressive strength of concrete.

## 2. Research Significance

This study aimed to forecast the compressive strength of concrete exposed to high temperatures by employing individual and ensemble machine learning algorithms. The decision tree (DT) and artificial neural network (ANN) (as a system), as well as the bagging regressor and gradient boosting regressor (as ensemble machine learning approaches) were used. The novelty of this research involves the investigation of the accuracy level of individual and ensemble ML algorithms, as well as the evaluation of the accuracy level of each approach for predicting the compressive strength of concrete at high temperatures. This study also compares statistical indicators that are used to evaluate the model’s accuracy. This study shows that the ensemble algorithms yielded a strong relationship when compared to individual machine learning techniques. Furthermore, the validity and accuracy of all the employed models were evaluated by using the method of k-fold cross-validation and by applying statistical checks. However, sensitivity analysis provides information regarding the contribution of the temperature parameter for predicting compressive strength. The purpose of this research also includes the comparison of the employed machine learning approaches with the techniques adopted in the literature.

## 3. Methodology

### 3.1. Supervised Machine Learning (ML) Techniques

Machine learning algorithms are more commonly applied in civil engineering for predicting the mechanical properties of concrete. Examples of their application are listed in the Table 1. The compressive or flexural strength of concrete can be determined by using the hit and trial method for various ages of concrete samples. To overcome some limitation in this method, we used machine learning algorithms to forecast outcomes for input data. Hao et al. [43] used the support vector machine (SVM) and k-fold cross-validation to predict the compressive strength of concrete in a marine environment, stating that the SVM performs better when compared to the artificial neural network (ANN) and decision tree (DT). Chengyeo et al. [44] predicted the compressive strength of concrete in a wet–dry environment using the backpropagation artificial neural network (BP-ANN). It was shown that the BP-ANN provides better accuracy regarding the actual and predicted results. Hocine et al. [45] applied the ANN model for predicting the compressive strength of limestone filler concrete. The training, testing, and validation of their data provides a strong correlation (exceeding 97%) with the real data. Behfernia et al. [46] used the ANN and adaptive neuro-based fuzzy inference (ANFIS) to predict the compressive strength of concrete. It was evaluated that the ANN model is a well-organized model for predicting the compressive strength of concrete. Hoang et al. [47] employed efficient machine learning models for predicting the strength of concrete. They proposed that the performance of the trained models of the gradient boosting regressor (GBR) and extreme gradient boosting (XGBoost) were better when compared to the support vector regressor and multilayer perceptron (MLR).

### 3.2. Description of the Obtained Data

**Figure 1:**
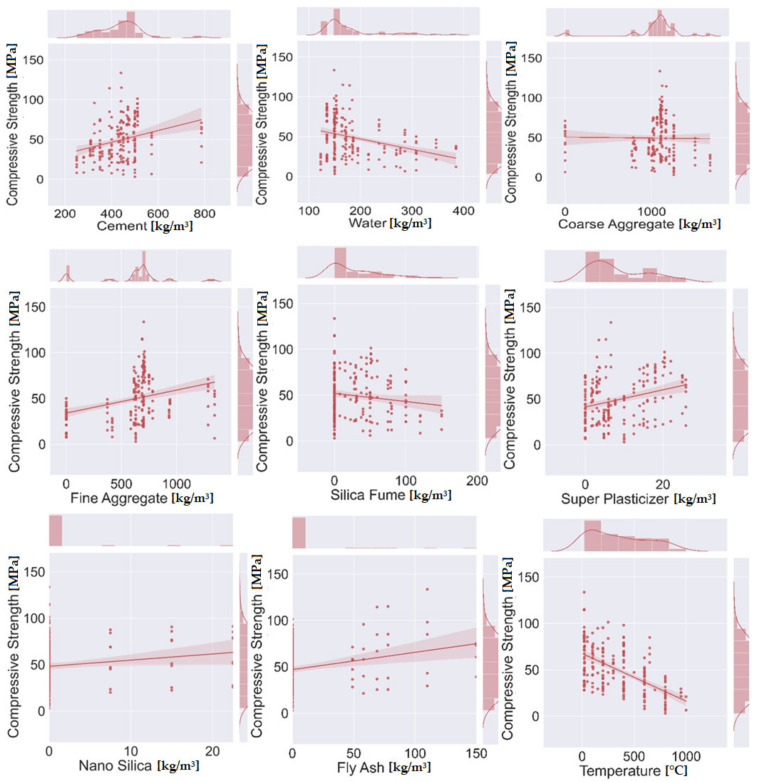
Contour plots showing the relative distribution of the parameters.

The data points used to run the models via machine learning algorithms were obtained from the literature [20,70,71,72,73,74,75,76,77], and can be seen in Appendix A. The data taken from the published article explains the behavior of concrete in a hot environment. Nine parameters were taken as the input parameters, namely, cement, water, fine aggregate, coarse aggregate, fly ash, superplasticizer, nano silica, silica fume, and temperature, while compressive strength was taken as the output parameter. These parameters were employed in Jupiter python software in order to indicate the graphical representation in the form of their relative frequency distributions, which can be seen in Figure 1. It is clear that the model’s performance was significantly affected by the input variables. The descriptive analysis, as well as the mathematical indication of the variables used to run the models (with their ranges), are listed in Table 2.

### 3.3. Machine Learning Approaches

**Figure 2:**
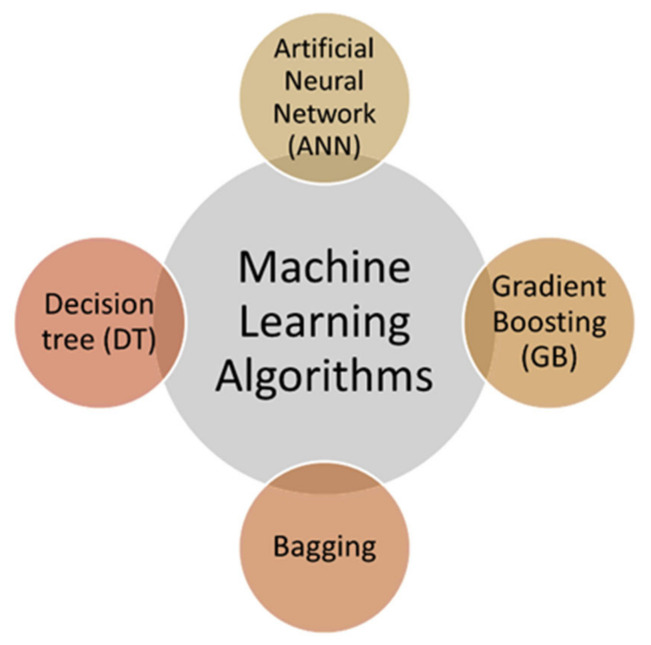
Machine learning techniques used in the research.

This section explains the types of algorithms used for predicting the compressive strength of concrete at high temperatures. The strength property (compressive strength) of concrete was forecasted using both ensemble and individual algorithms. The decision tree, bagging, and gradient boosting techniques were used to run the models. Python coding was used in Anaconda software for all three employed machine learning approaches. The applied algorithms are illustrated in Figure 2.

A decision tree is a supervised machine learning technique used for the distribution of regression problems, as well as for the classification of problems. The structure of the decision tree is like a flowchart with nodes, branches, and roots. The internal node exhibits a test on an attribute; every branch shows the outcome of the test, while each leaf node provides the indication of the class tag. The classification rule is represented by the path followed from the root to the leaf. Three different types of nodes of decision tree, with three geometric shapes (square, circle, and triangle), are available. It can generally be seen as a simple technique that can be used for understanding and interpreting.

Bagging is also known as bootstrap aggregating, the arrangement of bagging in such a way that can improve the firmness and accuracy of the machine learning algorithms used in the regression and classification. It is normally used to reduce the variances among the actual and predicted results. Bagging can be applied to any type of method but has commonly been applied with decision tree methods. It is also considered to be one of the special cases of the model averaging technique. Bagging is a parallel ensemble machine learning approach that gives an explanation about the variance of predicted models by providing supplementary data in the training stage. There are equal chances for each element to appear in the new dataset. Predictive power cannot be improved while altering the training set. The decision tree with bagging is modulated with 20 sub-models to have an optimized value, and as a result a strong adamant output result can be obtained.

Gradient boosting is generally considered and accepted as one of the powerful approaches for creating predictive models. It is an ensemble machine learning algorithm that is normally employed for regression and classification problems. It develops a forecasted model in the form of an ensemble of frail predicted models—normally the decision tree. When the decision tree provides the result as a weak learner, the resulting algorithm will then be considered as a gradient boosting tree. Gradient boosting can also be employed in the field of learning to rank. It is also used for high energy physics in data analysis.

The artificial neural network (ANN) algorithm has a brain-like structure with connected neurons. The ANN is essentially the collection of connected units or nodes (known as artificial neurons), which act as the model of the human brain. These neural networks learn by example of processing. They contain a known “input” and “result”, which creating probability-weighted associations among the input and result and are stored within the data structure of the net itself. The application of the ANN in the field of civil engineering is of great interest nowadays, especially for predicting the mechanical properties of concrete. This is due to its high accuracy level of predicting results for the actual strength properties of concrete.

## 4. Result and Analysis

### 4.1. Statistical Analysis

**Figure 3:**
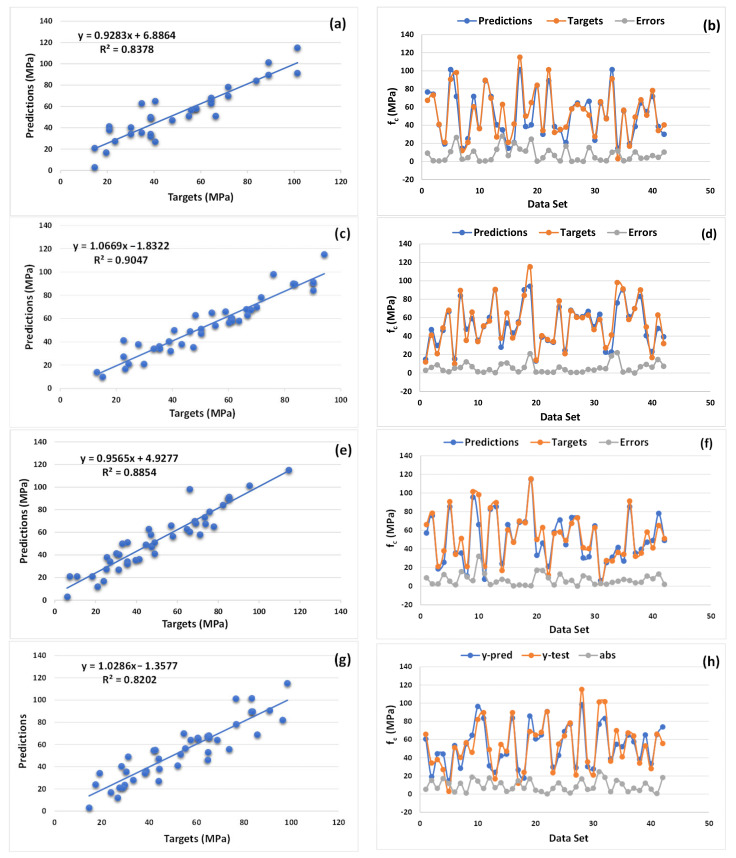
Numerical analysis results showing the relationship between the actual and predicted results, including the error distribution of models: DT (**a**,**b**); bagging (**c**,**d**); GB (**e**,**f**), ANN (**g**,**h**).

The statistical results for the actual and predicted (using supervised machine learning algorithms) compressive strength of concrete obtained at high temperature, as well as their error distribution, are shown in Figure 3. The accuracy level of the performance of the model was compared with the value of the correlation coefficient (R^2^). The DT (individual algorithm) model appeared to be better, with the value of R^2^ equal to 0.83, as depicted in Figure 3a. The model’s error distribution can be seen in Figure 3b. The minimum and maximum error values of the DT model were determined at a level of 14.5 MPa and 101.4 MPa, respectively. The average value of the errors was 51.2 MPa. However, 50% data of the errors data lay between 30 and 70 MPa, and only 7.1% data showed as error above 100 MPa, as illustrated in Figure 3b.

The predictive performance of the bagging (ensemble algorithm) model indicates a strong relation with the actual outcomes. The highest value of R^2^ (0.90) was obtained in the case of the bagging regressor. In turn, the values of R^2^ for the ANN, DT, and GB were equal to 0.82, 0.83, and 0.88, respectively. These results indicate a high accuracy level of the prediction. The graphical representation of the predicted and actual results of the compressive strength of concrete at high temperatures can be seen in Figure 3c, with its error distribution in Figure 3d. The maximum and minimum error values for the bagging regressor when predicting the strength property of concrete at increased temperatures were equal to 94.1 and 12.95 MPa, respectively. However, 59.92% of the errors data lay between 30 and 70 MPa, as shown in Figure 3d.

The gradient boosting (ensemble ML approach) model also indicates a better accuracy in the case of the predictive and actual outcomes for the compressive strength of concrete at high temperatures. In comparison, the performance of gradient boosting was almost similar to the bagging regressor (with less margin for the bagging regressor due to the R^2^ value being equal to 0.88), as shown in Figure 3e. The error distribution is shown in Figure 3f. The average value of the gradient boosting regressor was equal to 50.76 MPa, whereas the maximum and minimum error values were 114.5 and 6 MPa, respectively. In addition, only 4.76% of the error data were above 100 MPa for the regressor.

The same statistical result for the ANN model also indicates the better performance of this model when compared to the DT algorithm. The ANN model indicated a strong relation, with a smaller variance between the actual and predicted outcome, and provided the R^2^ value equal to 0.82, as shown in Figure 3g. The distribution of the errors for the ANN model can be seen in Figure 3h. The distribution indicates the maximum and minimum values of the error, which were equal to 24.58 and 0.29 MPa, respectively. However, the average value was equal to 9.158 MPa. It was also noted that 57.14% of the error data lay between 0 to 10 MPa, and 19.04% of the data lay between 10 to 15 MPa, with only 2.38% of the data being above 20 MPa.

### 4.2. k-Fold Cross Validation and Statistical Checks

To evaluate the model’s authentic execution, we adopted the k-fold cross validation approach. This method is normally employed to analyze the actual performance of models. In this test, the data were arranged randomly and divided into 10 groups. Nine groups were allocated for training purposes, and the remaining one was assigned for validation of the model. The average value was obtained by repeating the same process 10 times. The application of the 10-fold cross validation test was used to obtain the most accurate performance of the models. It was also important to apply the statistical checks in order to obtain the performance level of the model. This research also includes the application of the statistical check of the performance of the models with regards to the prediction according to Equations (1)–(5)

(1) $$\mathrm{RMSE}=\sqrt{\frac{\sum_{i=1}^{n}\;{\left(ex_{i}-mo_{i}\right)}^{2}}{n}}$$

(2) $$\mathrm{MAE}=\frac{\sum_{i=1}^{n}|ex_{i}-mo_{i}|}{n}$$

(3) $$\mathrm{RSE}=\frac{\sum_{i=1}^{n}{\left(mo_{i-}ex_{i}\right)}^{2}}{\sum_{i=1}^{n}{\left(\bar{ex}-ex_{i}\right)}^{2}}$$

(4) $$\mathrm{RRMSE}=\frac{1}{e}\sqrt{\frac{\sum_{i=1}^{n}{\left(ex_{i}-mo_{i}\right)}^{2}}{n}}$$

(5) $$R=\frac{\sum_{i=1}^{n}\left(ex_{i}-{\bar{ex}}_{i}\right)\left(mo_{i}-{\bar{mo}}_{i}\right)}{\sqrt{\sum_{i=1}^{n}{\left(ex_{i}-{\bar{ex}}_{i}\right)}^{2}\sum_{i=1}^{n}{\left(mo_{i}-{\bar{mo}}_{i}\right)}^{2}}}$$

where

$ex_{i}$ = the experimental value;
$mo_{i}$ = the predicted value;
${\bar{ex}}_{i}$ = the mean experimental value;
${\bar{mo}}_{i}$ = the mean predicted value obtained by the model;
*n* = the number of samples.

**Figure 4:**
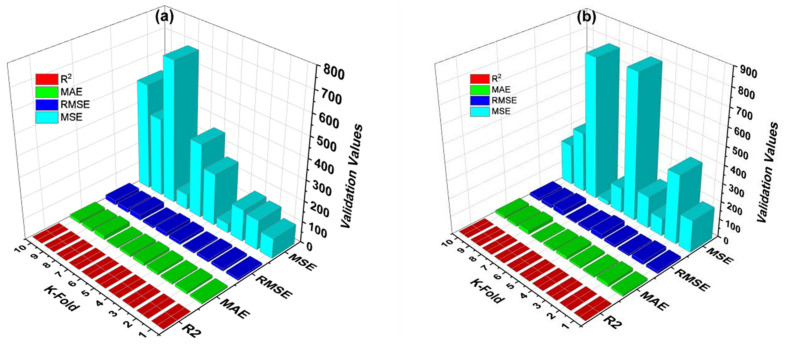

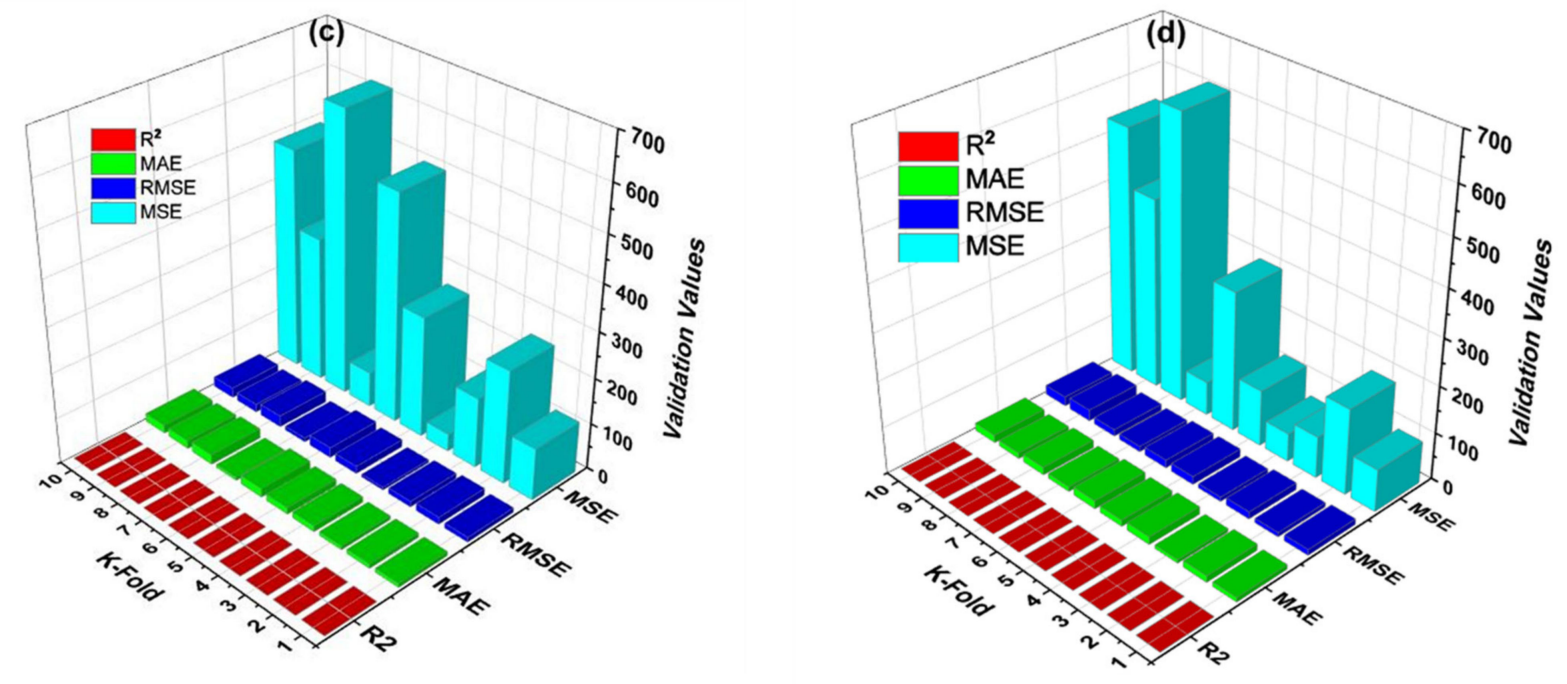
Statistical indication of the k-fold cross validation. DT (**a**); bagging (**b**); GB (**c**); ANN (**d**).

The correlation coefficient (R^2^), mean absolute error (MAE), mean square error (MSE), and root mean square error (RMSE) were introduced for evaluating the k-fold cross validation, as depicted in Figure 4. The validation process was performed for all the employed (DT, ANN, bagging, and gradient boosting) ML algorithms. The small values of the errors of the bagging model, and at the same time the increased value of the correlation coefficient (R^2^), indicated a better accuracy level when compared to the ANN, DT, and GB. The details of the analysis used for the k-fold cross validation process are included in Table 3.

In addition, the statistical checks, including mean absolute error (MAE), mean square error (MSE), and root mean square error (RMSE), were evaluated for all the machine learning approaches (Table 4). A smaller value of the error increased the value of the correlation coefficient (R^2^). The bagging regressor provided the value of MAE equal to 5.65 MPa, which was less than the MAE value of the DT (7.54 MPa), ANN (9.15 MPa), and GB (6.93 MPa). Similarly, the MSE and RMSE of the ANN was higher than the DT, bagging, and GB, while the R^2^ value of the ANN was lower than that of the other regressors.

Moreover, the statistical representation of the k-fold cross validation, including the correlation coefficient and errors, is presented in Figure 4. The average value of R^2^ for the DT was 0.42, with its minimum and maximum R^2^ values being equal to 0.03 and 0.82, respectively (Figure 4a)**.** The average R^2^ value of the bagging regressor was equal to 0.44, with its minimum and maximum R^2^ values being equal to 0.03 and 0.77 (Figure 4b). Similarly, the average R^2^ value of the gradient boosting was equal to 0.54, with its minimum and maximum values being 0.11 and 0.87, respectively (Figure 4c). The maximum and minimum values of R^2^ for the ANN were 0.84 and 0.037, respectively, while the average value was 0.42 (Figure 4d). The average values of each error (MAE, MSE, RMSE) for the DT were equal to 12.96, 269.79, and 15.26 MPa, respectively (Figure 4a); these average error values for the bagging were 13.64, 316.80, and 16.31 MPa, respectively (Figure 4b**)**. The same trend was also observed for the GB regressor, which had an average value of MAE equal to 13.79 MPa, with the values of MSE and RMSE being 282.08 and 15.72 MPa, respectively (as shown in Figure 4c). In addition, the average values of the errors (MAE, MSE, RMSE) for the ANN model were 13.44, 258.98, and 15.28 MPa, respectively (as presented in Figure 4d).

### 4.3. Sensitivity Analysis of the Compressive Strength of Concrete at High Temperatures

**Figure 5:**
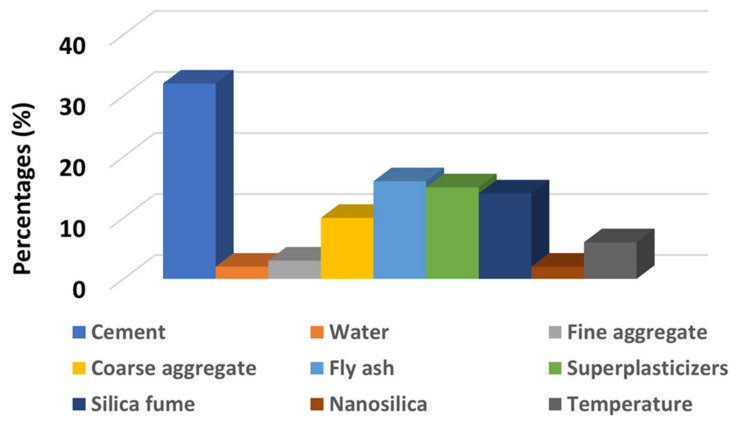
Bar chart indicating the performance of input parameters with regards to predicting of the compressive strength of concrete.

Sensitivity analysis was conducted in order to check the parameters that have a significant effect on the prediction of the compressive strength of concrete at high temperatures, as shown in Figure 5. Every variable used to run the model plays its role in predicting the strength of concrete. However, cement was the decisive factor that influenced the prediction of the strength of concrete. Its influence on the obtained results was estimated at 32%. In turn, the influence of fly ash, superplasticizers, silica fume, water, temperature, nano silica, fine aggregate, and coarse aggregate was estimated at the levels of 16%, 15%, 14%, 2%, 6%, 3%, 10%, and 2%, respectively. The result of the sensitivity analyses depends on the number of input parameters and the number of data points used to run the model. However, the contribution of each parameter is identified by the employed ML algorithm. The results of these analyses vary due to the different proportions of the concrete mix and the addition of new input parameters.

### 4.4. Discussion

This research shows a comparison of the performance of the various models with the experimental results of the compressive strength of concrete exposed to high temperatures. Ensemble (bagging, gradient boosting) and individual (ANN, DT) supervised machine learning algorithms were used for prediction purposes. The bagging regressor had a better prediction performance when compared to the ANN, DT, and GB. However, it is difficult to analyze and recommend the best machine learning regressor for predicting results for various topics because the performance of the models is directly affected by the input parameters and the data points used to run the model. However, ensemble machine learning techniques normally uses the weak learner by making the sub-models, which can be trained on data and uses the optimization to obtain a maximum value of R^2^. The performance of the 20 sub-models of the bagging and GB regressor, with their correlation coefficient (R^2^) values, can be seen in Figure 5. Thus, according to the literature, the performance of ensemble models shows more accurate results when compared to individual machine learning approaches. Previous studies also proven that the ensemble ML approaches such as bagging, boosting, and AdaBoost have better response towards the prediction of outcomes.

**Figure 6:**
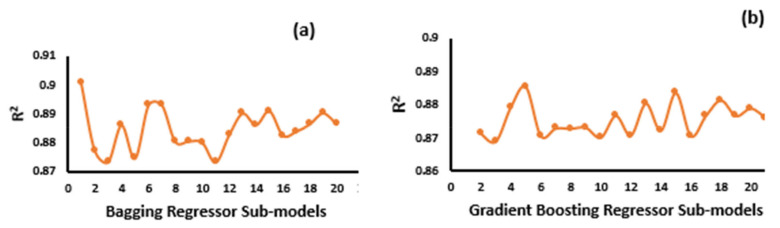
Sub-models representing the correlation coefficient (R^2^) values. Bagging (**a**); GB (**b**).

Moreover, it is also important to know about the performance of each parameter with regards to predicting outcomes. The sensitivity analysis provides information of how an individual parameter contributes towards the predicting of outcomes. The result of sensitivity analysis for this study can be seen in Figure 6. This study was also based on statistical checks, the validation process, and sensitivity analysis in order to verify the execution level of the evaluated ML techniques. This research could be beneficial with regards to reducing costs and minimizing the time consumed during the hit and trial method for achieving the desired strength of concrete. In addition, the research results can also be used in other fields of engineering for predicting required outcomes. It was shown that the ensemble modeling provides a better performance when compared to the other methods. Therefore, this technique is preferred for forecasting results in the case of related issues.

## 5. Conclusions and Future Recommendations

This research provides information about the predictive determination of the compressive strength of concrete at high temperatures using individual and ensemble supervised machine learning approaches. The application of the ML techniques for predicting the performance of concrete is quite an effective approach as it shows a high-level accuracy when compared to the actual result. It usually takes a large amount of time (28 days) to determine the strength of concrete. In turn, ML algorithms play an important role in reducing this time, and also save a large amount of the costs and efforts associated with the conducting of experimental works. In this research, the decision tree (DT) and ANN algorithms were selected from the individual techniques, while the bagging and gradient boosting (GB) regressors were used as ensemble algorithms for forecasting the strength of concrete at high temperatures. The bagging technique was most effective and had the highest correlation coefficient value. The lesser values of the errors (MAE of 5.65 MPa, MSE of 61.08 MPa, RMSE of 7.81) from the statistical checks for the bagging were also the indication of its better performance as opposed to ANN, DT, and GB. Practically, it is impossible to evaluate the effect of temperature on the mechanical properties of concrete prepared with various type of mixes. However, the temperature and other related effects such as humidity can also be added as input parameters for running the models to obtain the required output. The following conclusions form this study can be drawn: the ensemble algorithms (bagging and GB) performed well when predicting the compressive strength of concrete—not only at normal temperature, but also at high temperatures.

(a) The performance of the models can be affected by input parameters. Taking into account the thermal aspect (being the main consideration of the paper), we found that the ensemble models showed less discrepancy between actual and predicted results.
(b) The accuracy level of the bagging and GB regressors was also confirmed using the k-fold cross validation process.
(c) The contribution of each parameter with regards to predicting the outcome was evaluated by means of sensitivity analysis.
(d) This study describes the positive role of the supervised ML approaches in the field of civil engineering. The application of these techniques can be successfully adopted to predict the mechanical properties of concrete without spending time on the experimental work in the laboratory. It was also observed that the ensemble machine learning algorithms indicate a strong relation between actual and forecasted results when compared to individual algorithms.
(e) The high accuracy of the models can also be achieved by increasing the data points, as number of data points have high influence on the model’s outcome.
(f) The performance of the models can also be evaluated on the basis of practical work performed in a laboratory in order to understand the difference level between the actual and predicted result.
(g) The variance can be reduced by splitting more than 20 sub-models (in the ensemble techniques) for training on data and optimization would give the maximum R^2^ value.

It should be underlined that it is difficult to recommend or say about any approach directly on few trails that will provide the most accurate result, while the other techniques (such as AdaBoost Regressor) can be used for the prediction of outcomes for making comparisons.

## Figures and Tables

**Table 1:** Prediction properties using different approaches.

No.	Algorithm Used	Notation	Data Points	Prediction Properties	Year	Material Used	References
1.	Support vector machine	SVM	144	Compressive strength	2021	FA	[48]
2.	Gene expression programming	GEP	303	Bearing capacity of concrete-filled steel tube column	2019	_	[49]
3.	Data envelopment analysis	DEA	114	Compressive strength Slump test L-box test V-funnel test	2021	FA	[50]
4.	Gene expression programming, artificial neural network, decision tree	GEP, ANN, DT	642	Surface chloride concentration	2021	FA	[51]
5.	Support vector machine	SVM	-	Compressive strength	2020	FA	[52]
6.	Support vector machine	SVM	115	Slump test L-box test V-funnel test Compressive strength	2020	FA	[53]
7.	Gene expression programming	GEP	351	Compressive strength	2020	GGBS	[54]
8.	Gene expression programming	GEP	54	Compressive strength	2019	NZ (natural zeolite)	[54]
9.	Gene expression programming	GEP	357	Compressive strength	2020	-	[55]
10.	Random forest and gene expression programming	RF and GEP	357	Compressive strength	2020	-	[56]
11.	Artificial neuron network	ANN	205	Compressive strength	2019	FA GGBFS SF RHA	[57]
12.	Intelligent rule-based enhanced multiclass support vector machine and fuzzy rules	IREMSVM-FR with RSM	114	Compressive strength	2019	FA	[58]
13.	Random forest	RF	131	Compressive strength	2019	FA GGBFS FA	[59]
14.	Multivariate adaptive regression spline	M5 MARS	114	Compressive strength Slump test L-box test V-funnel test	2018	FA	[60]
15.	Random kitchen sink algorithm	RKSA	40	V-funnel test J-ring test Slump test Compressive strength	2018	FA	[61]
16.	Adaptive neuro fuzzy inference system	ANFIS	55	Compressive strength	2018	-	[62]
17.	Artificial neuron network	ANN	114	Compressive strength	2017	FA	[63]
18.	Artificial neuron network	ANN	69	Compressive strength	2017	FA	[64]
19.	Individual and ensemble algorithm	GEP, DT, and bagging	270	Compressive Strength	2021	FA	[42]
20.	Individual with ensemble modeling	ANN, bagging and boosting	1030	Compressive strength	2021	FA	[65]
21.	Multivariate	MV	21	Compressive strength	2020	Crumb rubber with SF	[66]
22.	Gene expression programming	GEP	277	Axial capacity	2020	-	[67]
23.	Adaptive neuro fuzzy inference system	ANFIS with ANN	7	Compressive strength	2020	POFA	[68]
24.	Response surface method, gene expression programming	RSM, GEP	108	Compressive strength	2020	Steel Fibers	[69]

**Table 2:** Descriptive analysis of the parameters.

Parameters Description	Cement	Water	Fine Aggregate	Coarse Aggregate	Fly Ash	Super Plasticizer	Silica Fume	Nano Silica	Temperature
Mean	437.69	182.92	610.13	1052.13	12.65	8.58	29.32	1.74	354.52
Standard error	6.64	4.16	22.06	21.51	2.30	0.53	2.58	0.36	19.99
Median	442.00	154.00	689.00	1110.00	0.00	6.00	7.50	0.00	300.00
Mode	500.00	150.00	0.00	1110.00	0.00	0.00	0.00	0.00	400.00
Standard deviation	95.49	59.90	317.39	309.41	33.07	7.60	37.09	5.25	287.65
Sample variance	9118.52	3588.58	100,736.87	95,735.94	1093.76	57.71	1375.35	27.54	82,743.10
Kurtosis	3.39	2.06	0.71	5.69	7.01	−0.62	1.02	8.45	−1.04
Skewness	0.98	1.67	−0.40	−1.97	2.74	0.74	1.26	3.08	0.47
Range	536.00	262.00	1345.00	1681.00	150.00	25.90	150.00	22.50	980.00
Minimum	250.00	123.00	0.00	0.00	0.00	0.00	0.00	0.00	20.00
Maximum	786.00	385.00	1345.00	1681.00	150.00	25.90	150.00	22.50	1000.00
Sum	90,601.00	37,864.80	126,297.00	217,790.00	2619.00	1776.20	6068.60	360.00	73,386.00
Count	207.00	207.00	207.00	207.00	207.00	207.00	207.00	207.00	207.00

**Table 3:** Analysis of the k-fold cross-validation.

	Decision Tree			Bagging				GB					ANN			
k-Fold	MAE	MSE	RMSE	R^2^	MAE	MSE	RMSE	R^2^	MAE	MSE	RMSE	R^2^	MAE	MSE	RMSE	R^2^
1.	8.65	96.27	9.81	0.76	12.26	178.85	13.37	0.48	8.94	113.11	10.64	0.75	9.75	90.48	12.48	0.83
2.	12.89	143.44	11.98	0.80	18.53	384.74	19.61	0.54	9.77	247.83	15.74	0.82	13.86	183.85	9.39	0.84
3.	8.28	153.70	12.40	0.70	8.17	97.45	9.87	0.77	8.47	153.30	12.38	0.57	7.50	128.58	15.39	0.62
4.	13.30	45.73	6.76	0.50	11.94	178.89	13.37	0.58	12.83	38.32	6.19	0.87	16.49	60.28	9.48	0.49
5.	15.80	250.80	15.84	0.82	18.60	806.46	28.40	0.38	14.22	265.39	16.29	0.79	13.59	199.39	17.49	0.76
6.	12.27	358.78	18.94	0.19	10.57	141.22	11.88	0.76	18.86	501.43	22.39	0.42	17.39	300.49	17.39	0.17
7.	7.04	75.20	8.67	0.12	3.66	23.85	4.88	0.47	7.01	76.66	8.76	0.11	10.49	72.48	11.94	0.15
8.	21.35	682.29	26.12	0.03	23.16	785.78	28.03	0.07	21.36	620.66	24.91	0.12	16.49	612.49	14.49	0.04
9.	15.15	378.37	19.45	0.29	14.23	339.29	18.42	0.03	18.24	318.46	17.85	0.31	12.49	409.38	24.49	0.27
10.	14.88	513.31	22.66	0.03	15.27	231.50	15.22	0.33	18.20	485.66	22.04	0.63	16.39	532.48	20.38	0.05

**Table 4:** Statistical checks.

Machine Learning Algorithms	MAE (MPa)	MSE (MPa)	RMSE (MPa)
Decision tree (DT)	7.54	112.23	10.79
Bagging	5.65	61.08	7.81
Gradient boosting (GB)	6.93	85.47	9.24
Artificial neural network (ANN)	9.15	121.66	11.03

## Data Availability

The data presented in this article are available within the article.

## Appendix A

**Table A1:** Data points used in modeling.

Cement (kg/m^3^)	Water (kg/m^3^)	Fine Aggregate (kg/m^3^)	Coarse Aggregate (kg/m^3^)	Fly Ash (kg/m^3^)	Super Plasticizer (kg/m^3^)	Silica Fume (kg/m^3^)	Nano Silica (kg/m^3^)	Temperature °C	Compressive Strength (MPa)
250	123	417	1681	0	0	0	0	20	28.16
250	123	417	1681	0	0	0	0	200	23.4
250	123	417	1681	0	0	0	0	400	18.57
250	123	417	1681	0	0	0	0	600	15.26
250	123	417	1681	0	0	0	0	800	8.01
350	172	373	1507	0	0	0	0	20	48.99
350	172	373	1507	0	0	0	0	100	44.58
350	172	373	1507	0	0	0	0	400	34.12
350	172	373	1507	0	0	0	0	600	24.41
350	172	373	1507	0	0	0	0	800	15.24
500	385	0	820	0	6	0	0	20	38
500	385	0	820	0	6	0	0	200	36
500	385	0	820	0	6	0	0	800	12
450	346.5	0	805	0	6	50	0	20	46
450	346.5	0	805	0	6	50	0	200	41.5
450	346.5	0	805	0	6	50	0	400	36.2
400	308	0	790	0	6	100	0	20	50
400	308	0	790	0	6	100	0	400	42
400	308	0	790	0	6	100	0	800	21
350	269.5	0	775	0	6	150	0	20	33
350	269.5	0	775	0	6	150	0	200	29
350	269.5	0	775	0	6	150	0	800	12.5
400	308	0	1038	0	4.8	0	0	20	32
400	308	0	1038	0	4.8	0	0	200	29.5
400	308	0	1038	0	4.8	0	0	400	28.5
360	277.2	0	1028	0	4.8	40	0	20	35
360	277.2	0	1028	0	4.8	40	0	400	29
360	277.2	0	1028	0	4.8	40	0	800	11
320	246.4	0	1015	0	4.8	80	0	20	38
320	246.4	0	1015	0	4.8	80	0	200	35
320	246.4	0	1015	0	4.8	80	0	800	12
280	215.6	0	1005	0	4.8	120	0	20	28
280	215.6	0	1005	0	4.8	120	0	200	27
280	215.6	0	1005	0	4.8	120	0	400	21
500	135	700	1110	0	14	30	0	20	82.47
500	135	700	1110	0	14	30	0	600	42.58
500	135	700	1110	0	14	30	0	800	22.03
500	135	700	1110	0	15	22.5	7.5	20	84.14
500	135	700	1110	0	15	22.5	7.5	400	68.99
500	135	700	1110	0	15	22.5	7.5	800	23.39
500	135	700	1110	0	16	15	15	20	85.84
500	135	700	1110	0	16	15	15	400	76.62
500	135	700	1110	0	16	15	15	800	25.28
500	135	700	1110	0	18	7.5	22.5	20	85.21
500	135	700	1110	0	18	7.5	22.5	400	79.12
500	135	700	1110	0	18	7.5	22.5	600	51.11
470	135	700	1110	0	16	60	0	20	87.38
470	135	700	1110	0	16	60	0	600	47.39
470	135	700	1110	0	16	60	0	800	18.82
470	135	700	1110	0	18	52.5	7.5	20	87.61
470	135	700	1110	0	18	52.5	7.5	400	68.94
470	135	700	1110	0	18	52.5	7.5	800	20.06
470	135	700	1110	0	20	45	15	20	90.6
470	135	700	1110	0	20	45	15	400	75.71
470	135	700	1110	0	20	45	15	600	51.12
470	135	700	1110	0	22	37.5	22.5	400	78.22
470	135	700	1110	0	22	37.5	22.5	600	52.49
470	135	700	1110	0	22	37.5	22.5	800	25.72
326	184	659	1124	58	3	0	0	20	95.8
326	184	659	1124	58	3	0	0	650	57.9
326	184	659	1124	58	3	0	0	800	40
326	184	659	1124	58	3	0	0	950	21.3
391	179	689	1172	69	3.5	0	0	20	114.4
391	179	689	1172	69	3.5	0	0	400	84.8
391	179	689	1172	69	3.5	0	0	800	36.8
391	179	689	1172	69	3.5	0	0	950	25.4
442	166	689	1125	78	5.3	0	0	20	115.1
442	166	689	1125	78	5.3	0	0	400	85.2
442	166	689	1125	78	5.3	0	0	650	73.5
442	166	689	1125	78	5.3	0	0	950	25.5
440	149	702	1099	110	6.6	0	0	20	133.6
440	149	702	1099	110	6.6	0	0	400	98.1
440	149	702	1099	110	6.6	0	0	650	84.9
440	149	702	1099	110	6.6	0	0	800	43.1
437	170	783	1016	49	1.9	0	0	300	57.2
437	170	783	1016	49	1.9	0	0	400	58
437	170	783	1016	49	1.9	0	0	500	47.2
437	170	783	1016	49	1.9	0	0	600	36.5
437	170	783	1016	49	1.9	0	0	700	28.3
500	150	630	1260	0	10	0	0	22	49
500	150	630	1260	0	10	0	0	300	41
500	150	630	1260	0	10	0	0	400	23
500	150	630	1260	0	10	0	0	600	8
500	150	630	1260	0	10	0	0	800	3
450	150	630	1260	0	10	50	0	22	52
450	150	630	1260	0	10	50	0	105	53
450	150	630	1260	0	10	50	0	400	27
450	150	630	1260	0	10	50	0	600	11
450	150	630	1260	0	10	50	0	800	6
425	150	630	1260	0	12.5	75	0	22	57
425	150	630	1260	0	12.5	75	0	105	66
425	150	630	1260	0	12.5	75	0	300	61
425	150	630	1260	0	12.5	75	0	600	21
425	150	630	1260	0	12.5	75	0	800	12
400	150	630	1260	0	15	100	0	22	64
400	150	630	1260	0	15	100	0	105	78
400	150	630	1260	0	15	100	0	300	65
400	150	630	1260	0	15	100	0	400	37
400	150	630	1260	0	15	100	0	800	21
308	185	933	968	0	6	0	0	20	37.5
308	185	933	968	0	6	0	0	100	31.5
308	185	933	968	0	6	0	0	150	29.4
308	185	933	968	0	6	0	0	200	29.2
308	185	933	968	0	6	0	0	250	34.7
310	186	940	976	0	7.7	31	0	20	44.5
310	186	940	976	0	7.7	31	0	50	44.3
310	186	940	976	0	7.7	31	0	150	46.5
310	186	940	976	0	7.7	31	0	200	48.9
310	186	940	976	0	7.7	31	0	250	47.1
512	154	711	1106	0	18	0	0	20	80.6
512	154	711	1106	0	18	0	0	50	80.5
512	154	711	1106	0	18	0	0	100	67.8
512	154	711	1106	0	18	0	0	200	78.9
512	154	711	1106	0	18	0	0	250	83.7
511	153	709	1122	0	20.4	51	0	20	85.1
511	153	709	1122	0	20.4	51	0	50	85.2
511	153	709	1122	0	20.4	51	0	100	89.6
511	153	709	1122	0	20.4	51	0	150	94.6
511	153	709	1122	0	20.4	51	0	250	101.3
500	150	750	1068	0	0	0	0	100	75.3
500	150	750	1068	0	0	0	0	200	68.9
500	150	750	1068	0	0	0	0	400	66
500	150	750	1068	0	0	0	0	600	35.4
350	150	750	1023	150	0	0	0	23	75.2
350	150	750	1023	150	0	0	0	200	73.3
350	150	750	1023	150	0	0	0	400	60.4
350	150	750	1023	150	0	0	0	600	39.2
475	150	750	1065	0	25	0	0	23	75.7
475	150	750	1065	0	25	0	0	100	75.4
475	150	750	1065	0	25	0	0	400	68.5
475	150	750	1065	0	25	0	0	600	34.2
390	195	585	1209	0	0	0	0	23	34.1
390	195	585	1209	0	0	0	0	100	35.6
390	195	585	1209	0	0	0	0	200	31.6
390	195	585	1209	0	0	0	0	600	16.8
390	195	585	1209	0	0	0	0	400	26.6
572	286	1345	0	0	0	0	0	600	43.4
786	236	1286	0	0	25.9	78.6	0	800	41.3
572	286	1345	0	0	0	0	0	23	58.3
572	286	1345	0	0	0	0	0	200	55
572	286	1345	0	0	0	0	0	400	52.2
572	286	1345	0	0	0	0	0	800	31.5
572	286	1345	0	0	0	0	0	1000	6.5
786	236	1286	0	0	25.9	78.6	0	23	71
786	236	1286	0	0	25.9	78.6	0	200	58
786	236	1286	0	0	25.9	78.6	0	400	65.4
786	236	1286	0	0	25.9	78.6	0	600	62.9
786	236	1286	0	0	25.9	78.6	0	1000	21
430	172	687	1030	0	1.6	0	0	20	61.8
430	172	687	1030	0	1.6	0	0	100	53.3
430	172	687	1030	0	1.6	0	0	200	55.5
430	172	687	1030	0	1.6	0	0	300	46.5
430	172	687	1030	0	1.6	0	0	600	20.6
441	164	653	1115	0	2.9	28	0	100	62.8
441	164	653	1115	0	2.9	28	0	200	64.7
441	164	653	1115	0	2.9	28	0	300	56.5
441	164	653	1115	0	2.9	28	0	600	21.8
495	149	615	1168	0	1.9	0	0	20	67.4
495	149	615	1168	0	1.9	0	0	200	59.7
495	149	615	1168	0	1.9	0	0	300	49
495	149	615	1168	0	1.9	0	0	600	21
465	149	615	1168	0	3.1	30	0	20	80.3
465	149	615	1168	0	3.1	30	0	100	68
465	149	615	1168	0	3.1	30	0	300	56.5
465	149	615	1168	0	3.1	30	0	600	23.4
450	149	615	1168	0	3.7	45	0	20	84.2
450	149	615	1168	0	3.7	45	0	100	70.8
450	149	615	1168	0	3.7	45	0	200	71.7
250	123	417	1681	0	0	0	0	100	25.74
350	172	373	1507	0	0	0	0	200	40.35
500	385	0	820	0	6	0	0	400	34.5
450	346.5	0	805	0	6	50	0	800	21
400	308	0	790	0	6	100	0	200	44
350	269.5	0	775	0	6	150	0	400	27
400	308	0	1038	0	4.8	0	0	800	7.5
360	277.2	0	1028	0	4.8	40	0	200	32
320	246.4	0	1015	0	4.8	80	0	400	30
280	215.6	0	1005	0	4.8	120	0	800	8.5
500	135	700	1110	0	14	30	0	400	69.87
500	135	700	1110	0	15	22.5	7.5	600	45.23
500	135	700	1110	0	16	15	15	600	48.79
500	135	700	1110	0	18	7.5	22.5	800	27.38
470	135	700	1110	0	16	60	0	400	69.86
470	135	700	1110	0	18	52.5	7.5	600	47.07
470	135	700	1110	0	20	45	15	800	22.32
470	135	700	1110	0	22	37.5	22.5	20	91.24
326	184	659	1124	58	3	0	0	400	69.2
391	179	689	1172	69	3.5	0	0	650	66.9
442	166	689	1125	78	5.3	0	0	800	37.9
440	149	702	1099	110	6.6	0	0	950	29.4
437	170	783	1016	49	1.9	0	0	20	71.2
500	150	630	1260	0	10	0	0	105	51
450	150	630	1260	0	10	50	0	300	49
425	150	630	1260	0	12.5	75	0	400	32
400	150	630	1260	0	15	100	0	600	28
308	185	933	968	0	6	0	0	50	37.2
310	186	940	976	0	7.7	31	0	100	44.1
512	154	711	1106	0	18	0	0	150	72.8
511	153	709	1122	0	20.4	51	0	200	95.3
500	150	750	1068	0	0	0	0	23	75.5
350	150	750	1023	150	0	0	0	100	73.7
475	150	750	1065	0	25	0	0	200	73.4
441	164	653	1115	0	2.9	28	0	20	73.9
495	149	615	1168	0	1.9	0	0	100	57.6
465	149	615	1168	0	3.1	30	0	200	69
450	149	615	1168	0	3.7	45	0	300	57.9
450	149	615	1168	0	3.7	45	0	600	22.6

## References

[1] Bigdeli Y., Barbato M. Use of a low-cost concrete-like fluorogypsum-based blend for applications in underwater and coastal protection structures. Proceedings of the OCEANS 2017—Anchorage Conference.

[2] Reiter L., Wangler T., Anton A., Flatt R.J. (2020). Setting on demand for digital concrete—Principles, measurements, chemistry, validation. Cem. Concr. Res..

[3] Amran Y.M., Alyousef R., Alabduljabbar H., El-Zeadani M. (2020). Clean production and properties of geopolymer concrete; A review. J. Clean. Prod..

[4] Dong B., Wang F., Abadikhah H., Hao L.Y., Xu X., Khan S.A., Wang G., Agathopoulos S. (2019). Simple fabrication of concrete with remarkable self-cleaning ability, robust superhydrophobicity, tailored porosity, and highly thermal and sound insulation. ACS Appl. Mater. Interfaces.

[5] Chica L., Alzate A. (2019). Cellular concrete review: New trends for application in construction. Constr. Build. Mater..

[6] Arora A., Almujaddidi A., Kianmofrad F., Mobasher B., Neithalath N. (2019). Material design of economical ultra-high performance concrete (UHPC) and evaluation of their properties. Cem. Concr. Compos..

[7] Farooq F., Akbar A., Khushnood R.A., Muhammad W.L.B., Rehman S.K.U., Javed M.F. (2020). Experimental investigation of hybrid carbon nanotubes and graphite nanoplatelets on rheology, shrinkage, mechanical, and microstructure of SCCM. Materials.

[8] Khan M., Ali M. (2019). Improvement in concrete behavior with fly ash, silica-fume and coconut fibres. Constr. Build. Mater..

[9] Ahmad W., Farooq S.H., Usman M., Khan M., Ahmad A., Aslam F., Al Yousef R., Al Abduljabbar H., Sufian M. (2020). Effect of coconut fiber length and content on properties of high strength concrete. Materials.

[10] Bostanci S.C. (2020). Use of waste marble dust and recycled glass for sustainable concrete production. J. Clean. Prod..

[11] Gupta S., Kua H.W., Pang S.D. (2020). Effect of biochar on mechanical and permeability properties of concrete exposed to elevated temperature. Constr. Build. Mater..

[12] Karahan O. (2017). Transport properties of high volume fly ash or slag concrete exposed to high temperature. Constr. Build. Mater..

[13] Hager I. (2013). Behaviour of cement concrete at high temperature. Bull. Pol. Acad. Sci. Tech. Sci..

[14] Khan M., Cao M., Chaopeng X., Ali M. (2021). Experimental and analytical study of hybrid fiber reinforced concrete prepared with basalt fiber under high temperature. Fire Mater..

[15] Pimienta P., Hager I. Mechanical Behaviour of HPC at High Temperature. https://www.researchgate.net/publication/293226828_Mechanical_properties_of_hpc_at_high_temperatures.

[16] Li L., Khan M., Bai C., Shi K. (2021). Uniaxial tensile behavior, flexural properties, empirical calculation and microstructure of multi-scale fiber reinforced cement-based material at elevated temperature. Materials.

[17] Noumowe A., Carre H., Daoud A., Toutanji H. (2006). High-strength self-compacting concrete exposed to fire test. J. Mater. Civ. Eng..

[18] Ma Q., Guo R., Zhao Z., Lin Z., He K. (2015). Mechanical properties of concrete at high temperature—A review. Constr. Build. Mater..

[19] Khan U.A., Jahanzaib H.M., Khan M., Ali M. (2018). Improving the tensile energy absorption of high strength natural fiber reinforced concrete with fly-ash for bridge girders. Key Eng. Mater..

[20] Husem M. (2006). The effects of high temperature on compressive and flexural strengths of ordinary and high-performance concrete. Fire Saf. J..

[21] Bodnarova L. (2013). Effect of high temperatures on cement composite materials in concrete structures. Acta Geodyn. Geomater..

[22] Tanyildizi H., Coşkun A. (2008). Performance of lightweight concrete with silica fume after high temperature. Constr. Build. Mater..

[23] Chan Y., Luo X., Sun W. (2000). Compressive strength and pore structure of high-performance concrete after exposure to high temperature up to 800 °C. Cem. Concr. Res..

[24] Janotka I., Nürnbergerová T., Nad L. (2000). Behaviour of high-strength concrete with dolomitic aggregate at high temperatures. Mag. Concr. Res..

[25] Kim J.-K., Han S.H., Song Y.C. (2002). Effect of temperature and aging on the mechanical properties of concrete: Part I. Experimental results. Cem. Concr. Res..

[26] Masaki K., Maki I. (2002). Effect of prolonged heating at elevated temperatures on the phase composition and textures of Portland cement clinker. Cem. Concr. Res..

[27] Memon S.A., Shah S.F.A., Khushnood R.A., Baloch W.L. (2019). Durability of sustainable concrete subjected to elevated temperature – A review. Constr. Build. Mater..

[28] Handoo S., Agarwal S. (2002). Physicochemical, mineralogical, and morphological characteristics of concrete exposed to elevated temperatures. Cem. Concr. Res..

[29] An J., Mikhaylov A., Richter U.H. (2020). Trade war effects: Evidence from sectors of energy and resources in Africa. Heliyon.

[30] An J., Mikhaylov A. (2020). Russian energy projects in South Africa. J. Energy S. Afr..

[31] Nguyen K.T., Navaratnam S., Mendis P., Zhang G.K., Barnett J., Wang H. (2020). Fire safety of composites in prefabricated buildings: From fibre reinforced polymer to textile reinforced concrete. Compos. Part. B Eng..

[32] Naser M.Z., Seitllari A. (2020). Concrete under fire: An assessment through intelligent pattern recognition. Eng. Comput..

[33] Cao M., Li L., Yin H., Ming X. (2019). Microstructure and strength of calcium carbonate (CaCO3) whisker reinforced cement paste after exposed to high temperatures. Fire Technol..

[34] Roy T., Matsagar V. (2021). Mechanics of damage in reinforced concrete member under post-blast fire scenario. Structures.

[35] Malik M., Bhattacharyya S.K., Barai S.V. (2021). Microstructural changes in concrete: Postfire scenario. J. Mater. Civ. Eng..

[36] Nin Chan S.Y., Luo X., Sun W. (2000). Effect of high temperature and cooling regimes on the compressive strength and pore properties of high performance concrete. Constr. Build. Mater..

[37] Tanyildizi H., Coşkun A. (2008). The effect of high temperature on compressive strength and splitting tensile strength of structural lightweight concrete containing fly ash. Constr. Build. Mater..

[38] Chan S.Y.N., Peng G.-F., Chan J.K.W. (1996). Comparison between high strength concrete and normal strength concrete subjected to high temperature. Mater. Struct..

[39] Tang Y., Feng W., Feng W., Chen J., Bao D., Li L. (2021). Compressive properties of rubber-modified recycled aggregate concrete subjected to elevated temperatures. Constr. Build. Mater..

[40] Khan M.A., Zafar A., Farooq F., Javed M.F., Alyousef R., Alabduljabbar H., Khan M.I. (2021). Geopolymer Concrete Compressive Strength via Artificial Neural Network, Adaptive Neuro Fuzzy Interface System, and Gene Expression Programming With K-Fold Cross Validation. Front. Mater..

[41] Fang Z., Roy K., Chen B., Sham C.-W., Hajirasouliha I., Lim J.B. (2021). Deep learning-based procedure for structural design of cold-formed steel channel sections with edge-stiffened and un-stiffened holes under axial compression. Thin Walled Struct..

[42] Ahmad A., Farooq F., Niewiadomski P., Ostrowski K., Akbar A., Aslam F., Alyousef R. (2021). Prediction of compressive strength of fly ash based concrete using individual and ensemble algorithm. Materials.

[43] Ling H., Qian C., Kang W., Liang C., Chen H. (2019). Combination of support vector machine and K-fold cross validation to predict compressive strength of concrete in marine environment. Constr. Build. Mater..

[44] Liang C., Qian C., Chen H., Kang W. (2018). Prediction of compressive strength of concrete in wet-dry environment by BP artificial neural networks. Adv. Mater. Sci. Eng..

[45] Ayat H., Kellouche Y., Ghrici M., Boukhatem B. (2018). Compressive strength prediction of limestone filler concrete using artificial neural networks. Adv. Comput. Des..

[46] Behfarnia K., Khademi F. (2017). A comprehensive study on the concrete compressive strength estimation using artificial neural network and adaptive neuro-fuzzy inference system. Int. J. Optim. Civ. Eng..

[47] Nguyen H., Vu T., Vo T.P., Thai H.-T. (2021). Efficient machine learning models for prediction of concrete strengths. Constr. Build. Mater..

[48] Huang J., Sun Y., Zhang J. (2021). Reduction of computational error by optimizing SVR kernel coefficients to simulate concrete compressive strength through the use of a human learning optimization algorithm. Eng. Comput..

[49] Sarir P., Chen J., Asteris P.G., Armaghani D.J., Tahir M.M. (2021). Developing GEP tree-based, neuro-swarm, and whale optimization models for evaluation of bearing capacity of concrete-filled steel tube columns. Eng. Comput..

[50] Balf F.R., Kordkheili H.M., Kordkheili A.M. (2021). A new method for predicting the ingredients of Self-Compacting Concrete (SCC) including Fly Ash (FA) using Data Envelopment Analysis (DEA). Arab. J. Sci. Eng..

[51] Ahmad A., Farooq F., Ostrowski K., Śliwa-Wieczorek K., Czarnecki S. (2021). Application of novel machine learning techniques for predicting the surface chloride concentration in concrete containing waste material. Materials.

[52] Azimi-Pour M., Eskandari-Naddaf H., Pakzad A. (2020). Linear and non-linear SVM prediction for fresh properties and compressive strength of high volume fly ash self-compacting concrete. Constr. Build. Mater..

[53] Saha P., Debnath P., Thomas P. (2020). Prediction of fresh and hardened properties of self-compacting concrete using support vector regression approach. Neural Comput. Appl..

[54] Shahmansouri A.A., Bengar H.A., Jahani E. (2019). Predicting compressive strength and electrical resistivity of eco-friendly concrete containing natural zeolite via GEP algorithm. Constr. Build. Mater..

[55] Aslam F., Farooq F., Amin M.N., Khan K., Waheed A., Akbar A., Javed M.F., Alyousef R., Alabdulijabbar H. (2020). Applications of gene expression programming for estimating compressive strength of high-strength concrete. Adv. Civ. Eng..

[56] Farooq F., Amin M.N., Khan K., Sadiq M.R., Javed M.F.F., Aslam F., Alyousef R. (2020). A comparative study of random forest and genetic engineering programming for the prediction of compressive strength of High Strength Concrete (HSC). Appl. Sci..

[57] Asteris P.G., Kolovos K. (2019). Self-compacting concrete strength prediction using surrogate models. Neural Comput. Appl..

[58] Selvaraj S., Sivaraman S. (2019). Prediction model for optimized self-compacting concrete with fly ash using response surface method based on fuzzy classification. Neural Comput. Appl..

[59] Zhang J., Ma G., Huang Y., Sun J., Aslani F., Nener B. (2019). Modelling uniaxial compressive strength of lightweight self-compacting concrete using random forest regression. Constr. Build. Mater..

[60] Kaveh A. (2017). M5′ and Mars based prediction models for properties of self-compacting concrete containing fly ash. Period. Polytech. Civ. Eng..

[61] Sathyan D., Anand K.B., Prakash A.J., Premjith B. (2018). Modeling the fresh and hardened stage properties of self-compacting concrete using random kitchen sink algorithm. Int. J. Concr. Struct. Mater..

[62] Vakhshouri B., Nejadi S. (2018). Prediction of compressive strength of self-compacting concrete by ANFIS models. Neurocomputing.

[63] Douma O.B., Boukhatem B., Ghrici M., Tagnit-Hamou A. (2016). Prediction of properties of self-compacting concrete containing fly ash using artificial neural network. Neural Comput. Appl..

[64] Abu Yaman M., Elaty M.A., Taman M. (2017). Predicting the ingredients of self compacting concrete using artificial neural network. Alex. Eng. J..

[65] Farooq F., Ahmed W., Akbar A., Aslam F., Alyousef R. (2021). Predictive modeling for sustainable high-performance concrete from industrial wastes: A comparison and optimization of models using ensemble learners. J. Clean. Prod..

[66] Bušić R., Benšić M., Miličević I., Strukar K. (2020). Prediction models for the mechanical properties of self-compacting concrete with recycled rubber and silica fume. Materials.

[67] Javed M.F., Farooq F., Memon S.A., Akbar A., Khan M.A., Aslam F., Alyousef R., Alabduljabbar H., Rehman S.K.U. (2020). New prediction model for the ultimate axial capacity of concrete-filled steel tubes: An evolutionary approach. Crystals.

[68] Al-Mughanam T., Aldhyani T., AlSubari B., Al-Yaari M. (2020). Modeling of compressive strength of sustainable self-compacting concrete incorporating treated palm oil fuel ash using artificial neural network. Sustainability.

[69] Nematzadeh M., Shahmansouri A.A., Fakoor M. (2020). Post-fire compressive strength of recycled PET aggregate concrete reinforced with steel fibers: Optimization and prediction via RSM and GEP. Constr. Build. Mater..

[70] Ergün A., Kürklü G., Serhat B.M., Mansour M.Y. (2013). The effect of cement dosage on mechanical properties of concrete exposed to high temperatures. Fire Saf. J..

[71] Cülfik M.S., Özturan T. (2010). Mechanical properties of normal and high strength concretes subjected to high temperatures and using image analysis to detect bond deteriorations. Constr. Build. Mater..

[72] Behnood A., Ziari H. (2008). Effects of silica fume addition and water to cement ratio on the properties of high-strength concrete after exposure to high temperatures. Cem. Concr. Compos..

[73] Bastami M., Baghbadrani M., Aslani F. (2014). Performance of nano-silica modified high strength concrete at elevated temperatures. Constr. Build. Mater..

[74] Chen L., Fang Q., Jiang X., Ruan Z., Hong J. (2015). Combined effects of high temperature and high strain rate on normal weight concrete. Int. J. Impact Eng..

[75] Xiong Y., Deng S., Wu D. (2016). Experimental study on compressive strength recovery effect of fire-damaged high strength concrete after realkalisation treatment. Procedia Eng..

[76] Mousa M.I. (2017). Effect of elevated temperature on the properties of silica fume and recycled rubber-filled high strength concretes (RHSC). HBRC J..

[77] Fu Y.F., Wong Y.L., Poon C.S., Tang C.A. (2005). Stress-strain behaviour of high-strength concrete at elevated temperatures. Mag. Concr. Res..

